# *Albizia Adianthifolia*: Botany, Medicinal Uses, Phytochemistry, and Pharmacological Properties

**DOI:** 10.1155/2018/7463584

**Published:** 2018-09-20

**Authors:** Alfred Maroyi

**Affiliations:** Medicinal Plants and Economic Development (MPED) Research Centre, Department of Botany, University of Fort Hare, Private Bag X1314, Alice 5700, South Africa

## Abstract

The bark, leaves, and roots of* Albizia adianthifolia* are highly sought after in tropical Africa as herbal medicines. Therefore, the aim of this study was to review the botany, medicinal uses, phytochemistry, and pharmacological properties of* A. adianthifolia* so as to provide baseline data required for evaluating the therapeutic potential of the species. Information on the botanical profile, medicinal uses, phytochemistry, and pharmacological properties of* A. adianthifolia* was undertaken using databases such as ScienceDirect, SciFinder, Pubmed, Google Scholar, Medline, SCOPUS, EThOS, ProQuest, OATD, and Open-thesis. Preelectronic literature search of conference papers, scientific articles, books, book chapters, dissertations, and theses was carried out at the University library. Literature search revealed that* A. adianthifolia* is used as purgative and herbal medicine for diabetes, eye problems, gastrointestinal problems, haemorrhoids, headache, neurodegenerative disorders, reproductive problems in women, respiratory problems, wounds and pain, skin diseases, sexually transmitted infections, and ethnoveterinary medicine. Phytochemical compounds identified from the species include apocarotenoids, chalcone, dipeptide, elliptosides, essential oils, fatty acids, flavonoids, histamine, imidazolyl carboxylic acid, prosapogenins, steroids, triterpene saponins, and triterpenoids. Pharmacological studies revealed that* A. adianthifolia* extracts and compounds have acetylcholinesterase enzyme inhibitory, anthelmintic, antiamoebic, antibacterial, antimycobacterial, anti-sexually transmitted infections, antifungal, anti-inflammatory, antioxidant, anxiolytic, and antidepressant, cognitive-enhancing, haemolytic, hypoglycemic and antihyperglycemic, immunomodulatory, and cytotoxicity activities. Detailed studies on the pharmacokinetics, in vivo, and clinical research involving compounds isolated from* A. adianthifolia* and extracts of the species are required.

## 1. Introduction

**Figure 1:**
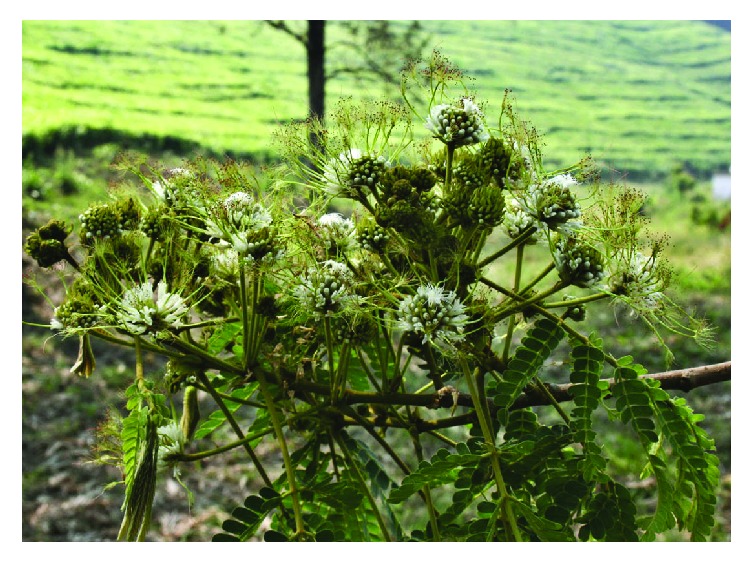
*Albizia adianthifolia*, a branch showing leaves and flowers (photo: MA Hyde).

*Albizia adianthifolia* (Schumach.) W. Wight is a medium to large tree (Figure 1) which belongs to the plant family Fabaceae and subfamily Mimosoideae. The species is a member of* Albizia* Durazz., a genus that is recognized worldwide for its high ecological, economical, and medicinal value [1].* Albizia* species have been used in folk medicine for the treatment of cough, diarrhoea, insomnia, irritability, rheumatism, stomach ache, tuberculosis, and wounds [2]. Phytochemical studies done on different species of* Albizia* lead to the isolation of different classes of secondary metabolites, such as saponins, terpenes, alkaloids, and flavonoids [2, 3]. The saponin compounds isolated from the genus* Albizia *have been reported to possess cancer related activities and other pharmacological properties which include analgesic, anthelmintic, antidysenteric, antihistaminic, anti-inflammatory, antimicrobial, antimutagenic, antiseptic, antispermatogenic, antitumour, anxiolytic, cytotoxic, immunomodulatory, nootropic, and apoptosis inducing properties [3]. According to Louppe et al. (2008) [1],* A. adianthifolia* is among 13* Albizia* species regarded as socially and economically important in tropical Africa as sources of high quality timber, gum, fodder, and herbal medicines. It is, therefore, not surprising that* A. adianthifolia* is considered as one of the most important African medicinal plants by Iwu [4] and, recently, Van Wyk [5] listed the bark of the species as commercially relevant medicinal and aromatic component of herbal medicines in Kenya and South Africa.* Albizia adianthifolia* is also included in the book “medicinal plants of South Africa”, a photographic guide to the most commonly used plant medicines in the country, including their botany, main traditional uses, and active ingredients. Research by Mander [6] showed that* A. adianthifolia* is ranked among the most frequently demanded medicinal plants in KwaZulu-Natal province in South Africa. Similarly, Williams et al. (2000, 2001) [7, 8] showed that the bark of the species is commonly traded in medicinal informal markets in Johannesburg, Gauteng province, South Africa. According to Williams et al. (2000) [7],* A. adianthifolia* was available in more than two-thirds (66%) of herbal medicine informal markets in Johannesburg. About 9050 kg to 10400 kg of* A. adianthifolia* bark were traded per annum as herbal medicine in 2001 in Gauteng province alone [9] and 21 200 kg traded per annum in KwaZulu-Natal province, South Africa [10–12]. Overcollection of* A. adianthifolia* bark as herbal medicine in KwaZulu-Natal province, South Africa, and the trade of its bark in informal herbal medicine markets in the country is causing a population decline and was identified as one of the 15 species becoming increasingly scarce in the province by urban herbalists [10, 13]. It is within this context that the current study was carried out aimed at reviewing the botany, medicinal uses, phytochemical, and pharmacological properties of* A. adianthifolia* so as to provide baseline data required for evaluating the therapeutic potential of the species.

## 2. Research Methodology

Search for information relevant to the botany, medicinal uses, phytochemical, and pharmacological properties of* A. adianthifolia* was carried out from October 2017 to May 2018. Online electronic databases including Google Scholar, SciFinder, ScienceDirect, Medline, Pubmed, SCOPUS, EThOS, ProQuest, OATD, and Open-thesis were used to search for relevant literature. Preelectronic literature of conference papers, scientific articles, books, book chapters, dissertations, and theses were carried out at the University of Fort Hare library. The keywords used in the electronic search criteria were “*Albizia adianthifolia*”, synonyms of the plant species “*A. chirindensis* (Swynn. ex Baker f.) Swynn. ex Steedman,* A. ealaensis* De Wild.,* A. fastigiata* (E. Mey.) Oliv.,* A. gummifera* auct. non (J. F. Gmel.) C.A. Sm.,* A. intermedia* De Wild. & T. Durand,* Inga fastigiata* (E. Mey.) Oliv.,* Mimosa adianthifolia* Schumach. and* Zygia fastigiata* E. Mey.”, English common names “flat-crown albizia, rough-bark flat-crown albizia and West African albizia”. The following keywords were used in combination with the species name, synonyms, and English common names to search for relevant information: “biological properties”, “ethnobotany”, “ethnomedicinal uses”, “ethnopharmacological properties”, “medicinal uses”, “pharmacological properties”, and “phytochemistry”. Publications included in this study were published between 1939 and 2018, including 60 articles published in international journals, books (13), conference, working papers and other scientific publications (eight), book chapters (three), dissertation, and website (one each). Three of the research articles were published before 1970, while four were published between 1970 and 1979, 1980 and 1989 (seven articles), 1990 and 1999 (10), 2000 and 2009 (32), and 2010 and 2010 (30 articles).

## 3. Botanical Profile and Description of* Albizia adianthifolia*

*Albizia* is a large genus comprising about 120 to 140 species that are widely distributed in tropical Africa (including Madagascar), central South America, south east Asia, and Malaysia [14]. The genus name* Albizia* was first published by Durazzini in 1772 based on a description of* A. julibrissin* Durazzin grown from seeds imported from Constantinople to Tuscany, Florence, in Italy by Fillippo Degli Albizzi in 1749 [15]. The species name “*adianthifolia*” refers to the resemblance of the leaves of the species to maidenhair fern, genus* Adiantum* L., family Pteridaceae [16, 17]. Literature studies revealed the existence of two accepted infraspecifics,* A. adianthifolia* var.* adianthifolia* [18, 19] and* A. adianthifolia* var.* intermedia* (De Wild. & T. Durand) Villiers [19, 20], and no attempt has been made to provide infraspecific circumscription and geographical distribution of the two varieties. Therefore,* A. adianthifolia sensu lato* will be used throughout this manuscript. Synonyms of* A. adianthifolia* include* A. chirindensis, A. ealaensis, A. fastigiata, A. gummifera, A. intermedia, Inga fastigiata, Mimosa adianthifolia,* and* Zygia fastigiata*.

**Figure 2:**
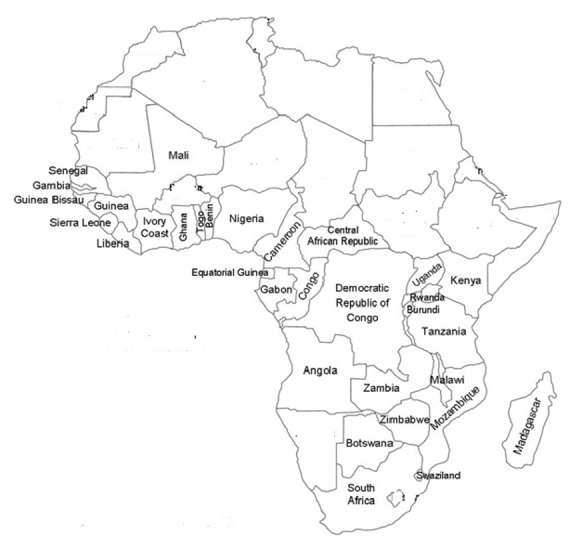
Distribution of* Albizia adianthifolia* in tropical Africa.

*Albizia adianthifolia* is a medium to large deciduous tree growing up to 35 m in height [16, 21]. The bole is up to 95 cm in diameter and is straight and cylindrical in closed forest but often crooked and/or twisted in more open savannah and bushland localities, usually without buttresses but with small, thick buttresses in forest localities [16]. The bark is yellowish brown to grey, smooth or rough, inner bark granular, creamy to yellowish in colour with clear gum.* Albizia adianthifolia* has a flattened crown, with large, spreading branches, young branches densely yellowish or reddish pubescent. Leaves are alternate, bipinnately compound with 3 to 10 pairs of pinnae with ovate to lanceolate stipules and leaflets in 5 to 17 pairs per pinna [16, 21]. The inflorescence is an axillary head with bisexual small flowers which are reddish to greenish white in colour. The fruit is an oblong, flat pod, densely but finely pubescent, transversely veined, and pale brown when ripe. The seeds are flattened, swollen, globose in shape and brown in colour [21].* Albizia adianthifolia* has been recorded in forests, woodlands, and areas that are transitional to woodland. The species occurs from South Africa through Madagascar, central and east Africa, to Senegal in the north (Figure 2).

The bark of* A. adianthifolia* is one of the most commonly stocked herbal medicine products in the informal herbal medicine markets in South Africa [7–13] and Grace et al. (2003) [22] tried to authenticate dried bark of the species using thin layer chromatography (TLC). This study showed that dried bark of* A. adianthifolia* is often confused with dried bark of* Acacia sieberiana* DC.,* Acacia xanthophloea* Benth. (family Fabaceae), and* Croton sylvaticus Hochst.* ex C. Krauss (family Euphorbiaceae), other three plant species sold as herbal medicines in the informal herbal medicine markets in South Africa. Grace et al. [22] argued that the notable similarities in the phytochemical fingerprints of* Acacia sieberiana, Acacia xanthophloea, A. adianthifolia,* and* Croton sylvaticus* may be an indicator of close usage relationships as similarities shown by TLC chromatograms may sometimes explain the phytochemical properties common to bark products that are purposefully substituted for one another, particularly in cases where taxonomically unrelated species are used [23].

## 4. Medicinal Uses of* Albizia adianthifolia*

**Figure 3:**
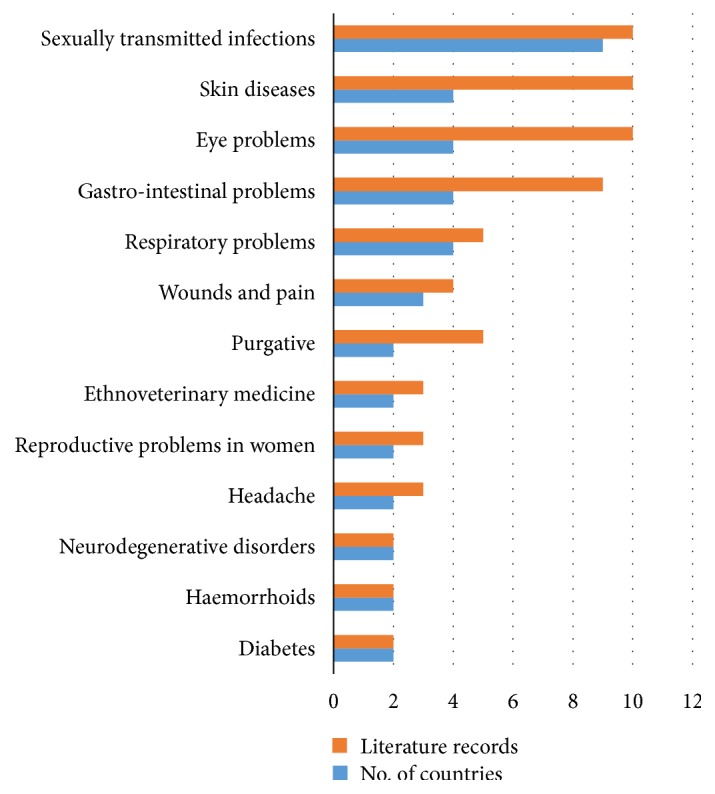
Major medicinal uses of* Albizia adianthifolia* in tropical Africa based on literature records.

The bark, leaf sap, leaves, roots, and stem bark of* A. adianthifolia* are used as remedies for human and animal diseases (Table 1). Ethnomedicinal uses of the species have been recorded in Burundi, Cameroon, the Democratic Republic of Congo (DRC), Guinea, Madagascar, Guinea-Bissau, Mozambique, Nigeria, Sierra Leone, Rwanda, Swaziland, South Africa, Tanzania, Uganda, Zimbabwe, and Togo, representing 51.6% of the countries where the species is indigenous (Figure 3). Major diseases and ailments recorded in at least two countries include diabetes, eye problems, gastrointestinal problems, haemorrhoids, headache, neurodegenerative disorders, purgative, reproductive problems in women, respiratory problems, wounds and pain, skin diseases, sexually transmitted infections, and ethnoveterinary medicine (Figure 3).* Albizia adianthifolia* is used to manage and treat top three ailments and diseases regarded by the World Health Organization [24] as the leading causes of death in low-income countries, and these are lower respiratory infections, diarrhoeal diseases, and ischaemic heart disease. The bark, leaves, and stem bark of* A. adianthifolia* are used as herbal remedies against bronchitis, cough, respiratory problems, and sinusitis in Cameroon, Mozambique, Nigeria, and South Africa [25–29], which can be categorized as the lower respiratory infections. The bark, leaves, and roots of* A. adianthifolia* are used as herbal remedies against diarrhoea, dysentery, and stomach ache in the DRC, Madagascar, Mozambique, South Africa, and Tanzania [26, 30–36]. The leaves of* A. adianthifolia* are used as herbal remedies against hypertension in Togo [37], which is one of the most common chronic diseases in modern societies. There is, therefore, a need for further research aimed at correlating some of the ethnomedicinal uses of* A. adianthifolia* to the phytochemical and biological activities of both the crude extracts and chemical compounds isolated from the species. Moreover, the World Health Organization has recognized the important role played by traditional medicines in the provision of primary healthcare in the resource-poor regions like tropical and subtropical Africa [38]. In addition to this, several studies have demonstrated the efficacy and importance of medicinal plants in the development of new pharmaceutical drugs and health products [39, 40].

Sexually transmitted infections are treated with multitherapeutic applications involving* A. adianthifolia* herbal concoctions. For example, in Sierra Leone, stem bark of* A. adianthifolia* is mixed with fruits of* Citrus aurantiifolia* (Christm.) Swingle and taken orally as herbal medicine for gonorrhoea [41]. In South Africa, the leaves of A. adianthifolia are mixed with the bark of* Trichilia dregeana* Sond. and taken orally as herbal medicine for syphilis [42]. In the Democratic Republic of Congo, leaves of* A. adianthifolia* are mixed with those of* Gynura scandens* O. Hoffm. and fruits of* Musa paradisiaca* L. and applied topically as herbal medicine for visible blisters on livestock [43].

## 5. Phytochemistry

**Figure 4:**
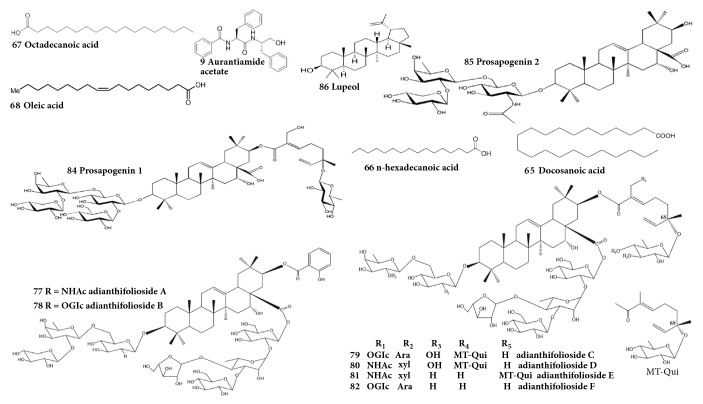
Chemical structures of some compounds isolated from* Albizia adianthifolia* that exhibited pharmacological activities.

To date, about 90 secondary metabolites have been isolated from the heartwood, leaves, roots, root, and stem bark of* A. adianthifolia*. The isolated phytochemical compounds which included apocarotenoids, dipeptide, elliptosides, essential oils, fatty acids, flavonoids, histamines, imidazolyl carboxylic acids, steroids, triterpene saponins, and triterpenoids were identified and characterized using fast atom bombardment mass spectroscopy (FABMS), gas chromatography-mass spectrometry (GC-MS), high performance liquid chromatography (HPLC), high-resolution electrospray ionisation mass spectroscopy (HRESIMS), and nuclear magnetic resonance (NMR) techniques (Table 2). The essential oils, fatty acids, triterpene saponins, flavonoids, and phenolics are considered the most prominent family of phytochemical compounds occurring in* A. adianthifolia* [27, 28, 63–71]. Research by Akande et al. (2018) [70] revealed that *β*-caryophyllene** 54** (23.0%), E-geranyl acetone** 7** (7.4%), acorenone** 38** (6.4%), viridiflorol** 48** (6.4%), *α*-zingiberene** 52** (6.3%), and ar-curcumene** 51** (4.6%) were the major constituents in the leaf oil, while essential oils** 54** (39.3%), selin-11-en-4-*α*-ol** 44** (10.4%), 53 (9.6%),** 51** (7.2%), caryophyllene oxide** 40** (6.4%), and *α*-humulene** 50** (5.6%) were the major constituents in the stem bark oil and essential oils** 54** (32.1%),** 44** (13.1%), 41 (8.4%), pentadecanal** 28** (6.1%), and** 50** (4.4%) were the major constituents in the root bark oil of* A. adianthifolia*. The gas chromatography-mass spectrometry analyses of n-hexane heartwood extract of* A. adianthifolia* resulted in the identification of n-hexadecanoic acid** 66 **(34.9%), stigmasterol** 75** (28.6%), oleic acid** 68** (6.3%), 24S,5*α* stigmast-7-en-3*β*-ol** 76** (4.4%), and chondrillasterol** 74** (18.2%), while 9,12-octadecadienoic acid (Z,Z), methyl ester** 64** (17.6%), and trans-13-octadecanoic acid, methyl ester** 69** (37.2%) were identified from the chloroform extract [68]. Candy et al. (1978) [64] and Beppe et al. (2014) [28] identified flavonoids 3-methoxyflavanone** 70**, apigenin** 71,** and melanoxetin** 72** from the heartwood and leaves of* A. adianthifolia*. Beppe et al. (2014) [28] estimated the total flavonoids in leaves of* A. adianthifolia* to be 0.53 ± 0.001 mg rutoside/g lyophilized powder, while the total phenolics in the leaves and stem bark of the species was estimated to be 1.5 to 30.2 *μ*g gallic acid equivalents/g dry weight [27, 28, 69]. Roques et al. (1977) [63] and Haddad et al. (2003, 2004) [66, 67] identified triterpene saponins as major phytochemical compounds in the roots and root bark of* A. adianthifolia*, and these included 16*α*-hydroxy-21*β*-[(2-hydroxybenzoyl)oxy]-3*β*-[(O-*β*-D-xylopyranosyl-(1→2)-O-*β*-D-fucopyranosyl-(1→6)-2-(acetylamino)-2-deoxy-*β*-D-glucopyranosyl)oxy]olean-12-en-28-oic acid 28-O-*α*-L-arabinofuranosyl-(1→4)-O-[*β*-D-glucopyranosyl-(1→3)]-O-*α*-L-rhamnopyranosyl-(1→2)-*β*-D-glucopyranosyl ester** 77**, 16*α*-hydroxy-21*β*-[(2-hydroxybenzoyl)-oxy]-3*β*-[(O-*β*-D-glucopyranosyl-(1→2)-O-[O-*β*-D-xylopyranosyl-(1→2)-O-*β*-D-fucopyranosyl-(1→6)]-*β*-D-glucopyranosyl)oxy]-olean-12-en-28-oic acid 28-O-*α*-L-arabinofuranosyl-(1→4)O-[*β*-D-glucopyranosyl-(1→3)]-O-*α*-L-rhamnopyranosyl-(1→2)*β*-D-glucopyranosyl ester** 78**, 3-O-{O-*α*-L-arabinopyranosyl-(1→2)-O-*β*-d-fucopyranosyl-(1→6)-O-[*β*-d-glucopyranosyl-(1→2)]-*β*-d-glucopyranosyl}-21-O-{(2E,6S)-6-{{4-O-[(2E,6S)-2,6-dimethyl-6-(*β*-D-quinovopyranosyloxy)octa-2,7-dienoyl]-*β*-d-quinovopyranosyl}oxy}-2-(hydroxymethyl)-6-methylocta-2,7-dienoyl}acacic acid 28-{O-*α*-L-arabinofuranosyl-(1→4)-O-[*β*-d-glucopyranosyl-(1→3)]-O-*α*-L-rhamnopyranosyl-(1→2)-*β*-d-glucopyranosyl} ester** 79**, 21-O-{(2E,6S)-6-{{4-O-[(2E,6S)-2,6-dimethyl-6-(*β*-d-quinovopyranosyloxy)octa-2,7-dienoyl]-*β*-d-quinovopyranosyl}oxy}-2-(hydroxymethyl)-6-methylocta-2,7-dienoyl}-3-O-{O-*β*-D-xylopyranosyl-(1→2)-O-*β*-d-fucopyranosyl-(1→6)-2-(acetylamino)-2-deoxy-*β*-d-glucopyranosyl}acacic acid 28-{O-*α*-L-arabinofuranosyl-(1→4)-O-[*β*-d-glucopyranosyl-(1→3)]-O-*α*-L-rhamnopyranosyl-(1→2)-*β*-d-glucopyranosyl} ester** 80**, 21-O-{(2E,6S)-6-{{3-O-[(2E,6S)-2,6-dimethyl-6-(*β*-d-quinovopyranosyloxy)octa-2,7-dienoyl]-*β*-d-quinovopyranosyl}oxy}-2,6-dimethylocta-2,7-dienoyl}-3-O-{O-*β*-D-xylopyranosyl-(1→2)-O-*β*-d-fucopyranosyl-(1→6)-2-(acetylamino)-2-deoxy-*β*-d-glucopyranosyl}acacic acid 28-{O-*α*-L-arabinofuranosyl-(1→4)-O-[*β*-d-glucopyranosyl-(1→3)]-O-*α*-L-rhamnopyranosyl-(1→2)-*β*-d-glucopyranosyl} ester** 81,** and 3-O-{O-*α*-L-arabinopyranosyl-(1→2)-O-*β*-d-fucopyranosyl-(1→6)-O-[*β*-d-glucopyranosyl-(1→2)]-*β*-d-glucopyranosyl}-21-O-{(2E,6S)-2,6-dimethyl-6-(*β*-d-quinovopyranosyloxy)octa-2,7-dienoyl}acacic acid 28-{O-*α*-L-arabinofuranosyl-(1→4)-O-[*β*-d-glucopyranosyl-(1→3)]-O-*α*-L-rhamnopyranosyl-(1→2)-*β*-d-glucopyranosyl} ester** 82** which have been shown to be cytotoxic against a large panel of cancer cells [3, 66]. Further comprehensive studies focusing on chemical constituents of* A. adianthifolia* and their pharmacological activities are required. Chemical structures of aurantiamide acetate** 9**, docosanoic acid** 65**, n-hexadecanoic acid** 66**, octadecanoic acid** 67**, oleic acid** 68**, 16*α*-hydroxy-21*β*-[(2-hydroxybenzoyl)oxy]-3*β*-[(O-*β*-D-xylopyranosyl-(1→2)-O-*β*-D-fucopyranosyl-(1→6)-2-(acetylamino)-2-deoxy-*β*-D-glucopyranosyl)oxy]olean-12-en-28-oic acid 28-O-*α*-L-arabinofuranosyl-(1→4)-O-[*β*-D-glucopyranosyl-(1→3)]-O-*α*-L-rhamnopyranosyl-(1→2)-*β*-D-glucopyranosyl ester** 77**, 16*α*-hydroxy-21*β*-[(2-hydroxybenzoyl)-oxy]-3*β*-[(O-*β*-D-glucopyranosyl-(1→2)-O-[O-*β*-D-xylopyranosyl-(1→2)-O-*β*-D-fucopyranosyl-(1→6)]-*β*-D-glucopyranosyl)oxy]-olean-12-en-28-oic acid 28-O-*α*-L-arabinofuranosyl-(1→4)O-[*β*-D-glucopyranosyl-(1→3)]-O-*α*-L-rhamnopyranosyl-(1→2)*β*-D-glucopyranosyl ester** 78**, 3-O-{O-*α*-L-arabinopyranosyl-(1→2)-O-*β*-d-fucopyranosyl-(1→6)-O-[*β*-d-glucopyranosyl-(1→2)]-*β*-d-glucopyranosyl}-21-O-{(2E,6S)-6-{{4-O-[(2E,6S)-2,6-dimethyl-6-(*β*-D-quinovopyranosyloxy)octa-2,7-dienoyl]-*β*-d-quinovopyranosyl}oxy}-2-(hydroxymethyl)-6-methylocta-2,7-dienoyl}acacic acid 28-{O-*α*-L-arabinofuranosyl-(1→4)-O-[*β*-d-glucopyranosyl-(1→3)]-O-*α*-L-rhamnopyranosyl-(1→2)-*β*-d-glucopyranosyl} ester** 79**, 21-O-{(2E,6S)-6-{{4-O-[(2E,6S)-2,6-dimethyl-6-(*β*-d-quinovopyranosyloxy)octa-2,7-dienoyl]-*β*-d-quinovopyranosyl}oxy}-2-(hydroxymethyl)-6-methylocta-2,7-dienoyl}-3-O-{O-*β*-D-xylopyranosyl-(1→2)-O-*β*-d-fucopyranosyl-(1→6)-2-(acetylamino)-2-deoxy-*β*-d-glucopyranosyl}acacic acid 28-{O-*α*-L-arabinofuranosyl-(1→4)-O-[*β*-d-glucopyranosyl-(1→3)]-O-*α*-L-rhamnopyranosyl-(1→2)-*β*-d-glucopyranosyl} ester** 80**, 21-O-{(2E,6S)-6-{{3-O-[(2E,6S)-2,6-dimethyl-6-(*β*-d-quinovopyranosyloxy)octa-2,7-dienoyl]-*β*-d-quinovopyranosyl}oxy}-2,6-dimethylocta-2,7-dienoyl}-3-O-{O-*β*-D-xylopyranosyl-(1→2)-O-*β*-d-fucopyranosyl-(1→6)-2-(acetylamino)-2-deoxy-*β*-d-glucopyranosyl}acacic acid 28-{O-*α*-L-arabinofuranosyl-(1→4)-O-[*β*-d-glucopyranosyl-(1→3)]-O-*α*-L-rhamnopyranosyl-(1→2)-*β*-d-glucopyranosyl} ester** 81** and 3-O-{O-*α*-L-arabinopyranosyl-(1→2)-O-*β*-d-fucopyranosyl-(1→6)-O-[*β*-d-glucopyranosyl-(1→2)]-*β*-d-glucopyranosyl}-21-O-{(2E,6S)-2,6-dimethyl-6-(*β*-d-quinovopyranosyloxy)octa-2,7-dienoyl}acacic acid 28-{O-*α*-L-arabinofuranosyl-(1→4)-O-[*β*-d-glucopyranosyl-(1→3)]-O-*α*-L-rhamnopyranosyl-(1→2)-*β*-d-glucopyranosyl} ester** 82**, acacic acid 3-O-beta-D-xylopyranosyl-(1-->2)-beta-D-fucopyranosyl-(1-->6)-2-acetylamino-2-deoxy-beta-D-glucopyranoside** 84**, acacic acid 3-O-(beta-D-xylopyranosyl-(1-->2)-beta-D-fucopyranosyl-(1-->6)- [beta-D-glucopyranosyl-(1-->2)]-beta-D-glucopyranosyl)-21-O-(6(S)-2-hydroxymethyl-6-methyl-6-O-(beta-D-quinovopyranosyl)-2,7-octadienoyl) ester** 85,** and lupeol** 86** which exhibited pharmacological properties [27, 65–68] are shown in Figure 4.

## 6. Pharmacological Activities

Over the years, pharmacological studies on* A. adianthifolia* extracts and compounds extracted from the species showed potent in vitro and in vivo pharmacological activities including acetylcholinesterase enzyme inhibitory [69, 72, 73], anthelmintic [70, 74], antiamoebic [74], antibacterial [27, 53, 68, 73, 75], antimycobacterial [76], anti-sexually transmitted infections [77], antifungal [27, 68], anti-inflammatory [73, 78], antioxidant [27, 28, 69, 79, 80], anxiolytic and antidepressant [79], cognitive-enhancing [28], haemolytic [66, 81], hypoglycemic and antihyperglycemic [45], immunomodulatory [66], cytotoxicity [77, 80–84].

## 7. Acetylcholinesterase Enzyme Inhibitory Activities

Risa et al. (2004) [72] evaluated the acetylcholinesterase inhibiting activities of aqueous and ethanol stem bark extracts of* A. adianthifolia* using thin layer chromatography (TLC) and microtitre plate assays. The aqueous and ethanol extracts yielded 14% and 8% inhibition at 0.1 mg/ml in the microplate assay and the ethanol extract exhibited weak inhibiting zone in the TLC assay [72]. Eldeen et al. (2005) [73] evaluated acetylcholinesterase enzyme inhibitory activities of ethanol and ethyl acetate bark and root extracts of* A. adianthifolia* using thin layer chromatography (TLC) and microplate assays with galanthamine as the positive control. The extracts exhibited moderate activities with percentage inhibition ranging from 45% to 61% and half maximal inhibitory concentration (IC_50_) values ranging from 0.4 mg/ml to 1.2 mg/ml; these values were lower than percentage inhibition of 93% and IC_50_ value of 2*μ*M exhibited by the control, galanthamine, at a concentration of 2*μ*M [73]. Sonibare et al. (2017) [69] evaluated the acetylcholinesterase inhibitory activities of methanol, ethyl acetate, chloroform, and n-hexane leaf extracts of* A. adianthifolia*. All extracts showed activities with IC_50 _values ranging from 10.0 *μ*g/mL to 124.4 *μ*g/mL [69]. The ability of* A. adianthifolia* extracts to inhibit acetylcholinesterase corroborates the traditional use of the species in the management of memory loss and neurodegenerative disorders in South Africa and Nigeria [32, 51].

## 8. Anthelmintic Activities

McGaw et al. (2000) [74] evaluated anthelmintic activities of hexane, ethanol, and water leaf extracts of* A. adianthifolia* on the mortality and reproductive ability of the free-living nematode* Caenorhabditis elegans* in two different assays. All extracts exhibited activities at both concentrations of 1 mg/ml and 2 mg/ml after two-hour and seven-day incubation periods [74]. Akande et al. (2018) [70] evaluated the anthelmintic activities of essential oils isolated from the leaves, root bark, and stem bark of* A. adianthifolia* using* Eudrilus eugeniae* adult earthworm with albendazole as the standard. The time of paralysis and death of* Eudrilus eugeniae* worms decreased as concentration was increased. The leaf essential oil showed the best activity with time of paralysis and death at 12.6 minutes and 60.2 minutes, respectively, which was higher than 82.8 minutes and 154.6 minutes exhibited by albendazole, the anthelmintic drug [70].

## 9. Antiamoebic Activities

McGaw et al. (2000) [74] evaluated antiamoebic activities of ethanol and water leaf extracts of* A. adianthifolia* using microdilution technique against the enteropathogenic* Entamoeba histolytica* with metronidazole as the positive control. The extracts showed weak activities with IC_50_ value of >5.0 mg/ml which was higher than 0.20 *μ*g/ml exhibited by metronidazole [74].

## 10. Antibacterial Activities

Van Puyvelde et al. (1983) [53] evaluated antibacterial activities of leaf extracts of* A. adianthifolia* against* Neisseria gonorrhoeae, Neisseria meningitidis, Streptococcus pyogenes,* and* Staphylococcus aureus* using the disk diffusion method. The extracts showed activities against* Neisseria gonorrhoeae* and* Neisseria meningitidis* with zone of inhibition ranging from 10 mm to 12 mm [53]. Eldeen et al. (2005) [73] evaluated antibacterial activities of aqueous, ethanol, and ethyl acetate bark and root extracts of A. adianthifolia against* Bacillus subtilis, Staphylococcus aureus, Micrococcus luteus, Escherichia coli,* and* Klebsiella pneumoniae* using the disc-diffusion and microdilution assays with neomycin (0.2 mg/ml) as the positive control. Ethanol bark extracts were active against all tested bacteria with minimum inhibitory concentration (MIC) values ranging from 3.13 mg/ml to 6.25 mg/ml, while ethyl acetate bark extract was active against all the pathogens except* Klebsiella pneumoniae* with MIC values ranging from 6.25 mg/ml to 12.5 mg/ml [73]. Abubakar and Majinda [68] evaluated antibacterial activities of chloroform and n-hexane extracts of heartwood of* A. adianthifolia* against* Escherichia coli, Pseudomonas aeruginosa, Bacillus subtilis *and* Staphylococcus aureus* using the modified agar overlay method with chloramphenicol as the positive control. The n-hexane and chloroform extracts showed the best activities against Escherichia coli with minimum inhibition quantity (MIQ) of 1 *μ*g each while other extracts exhibited moderate activities with MIQ value of 50 *μ*g and chloramphenicol exhibited activities with MIQ values ranging from 0.25 *μ*g to 10 *μ*g [68]. Tchinda et al. (2017) [75] evaluated antibacterial activities of methanol bark and root extracts of* A. adianthifolia* against* Pseudomonas aeruginosa, Klebsiella pneumoniae, Enterobacter aerogenes, Escherichia coli,* and* Providencia stuartii* using the broth microdilution assay. The extracts showed moderate to weak activities against tested pathogens with MIC values ranging from 128 *μ*g/mL to 1024 *μ*g/mL [75].

Tamokou et al. (2012) [27] evaluated the antibacterial activities of ethyl acetate extract, aurantiamide acetate** 9, **docosanoic acid** 65, **n-hexadecanoic acid** 66, **octadecanoic acid** 67, **oleic acid** 68,** and lupeol** 86** isolated from the stem bark of* A. adianthifolia* against* Enterococcus faecalis, Staphylococcus aureus, Pseudomonas aeruginosa, Escherichia coli, Klebsiella pneumoniae, Proteus mirabilis, Shigella flexneri,* and* Salmonella typhi* using the broth microdilution method with gentamicin as the positive control. The ethyl acetate extract and aurantiamide acetate** 9** were active against all the tested pathogens with MIC values ranging from 0.09 mg/ml to 0.78 mg/ml and 0.05 mg/ml to 0.1 mg/ml, respectively [27]. The compound lupeol** 86**, a mixture of n-hexadecanoic acid and oleic acid** 68**, and a mixture of compounds docosanoic acid** 65, **n-hexadecanoic acid** 66,** and octadecanoic acid** 67** were active against* Enterococcus faecalis, Staphylococcus aureus, Proteus mirabilis,* and* Shigella flexneri* with MIC values ranging from 0.1 mg/ml to 0.4 mg/ml, 0.05 mg/ml to 0.4 mg/ml, and 0.1 mg/ml to 0.8 mg/ml, respectively. The exhibited minimum bactericidal concentrations (MBC) were 0.39 mg/ml to 1.56 mg/ml for ethyl acetate extract, 0.1 mg/ml to 0.4 mg/ml (n-hexadecanoic acid** 66** and oleic acid** 68**), 0.2 mg/ml to 0.8 mg/ml (docosanoic acid** 65, **n-hexadecanoic acid** 66** and octadecanoic acid** 67**), 0.2 mg/ml to 0.4 mg/ml (compound lupeol** 86**), and 0.05 mg/ml to 0.1 mg/ml (aurantiamide acetate** 9**) [27]. The documented antibacterial activities exhibited by different extracts and compounds isolated from* A. adianthifolia* corroborate the traditional application of the species as herbal medicine against bacterial infections causing diarrhoea, dysentery, and stomachache in DRC, Madagascar, Mozambique, South Africa, and Tanzania [26, 30–36].

## 11. Antimycobacterial Activities

Eldeen and Van Staden [76] evaluated the antimycobacterial activities of dichloromethane, ethyl acetate, and ethanol bark and leaf extracts of* A. adianthifolia* against* Mycobacterium aurum* A+ using the broth microdilution method. Only the ethanol root extract exhibited moderate activity with MIC value of 6.3 mg/ml [76]. These findings show potential of* A. adianthifolia* in the treatment and management of respiratory problems such as bronchitis, cough, and sinusitis in Cameroon, Mozambique, Nigeria, and South Africa [25–29].

## 12. Anti-Sexually Transmitted Infections Activities

Naidoo et al. (2013) [77] evaluated anti-sexually transmitted infections activities of aqueous and dichloromethane and methanol (1:1) bark extracts of* A. adianthifolia* against* Candida albicans, Gardnerella vaginalis, Neisseria gonorrhoeae, Oligella ureolytica, Trichomonas vaginalis,* and* Ureaplasma urealyticum* using the microtitre plate dilution method with ciprofloxacin and amphotericin B as positive controls. The anti-sexually transmitted infections interaction of* A. adianthifolia* used in combination with* Trichilia dregeana* was determined by calculating the sum of the fractional inhibitory concentrations (∑FIC) against* Oligella ureolytica*. The extracts exhibited activities with MIC values ranging from 0.3 mg/mL to >16.0 mg/mL with average MIC value of 6.3 mg/mL while the controls, ciprofloxacin (0.01 mg/mL) and amphotericin B (0.1 mg/mL), exhibited MIC values of 0.04 *μ*g/mL to 0.6 *μ*g/mL and 2.5 *μ*g/mL, respectively [77]. The combination of* A. adianthifolia* with* Trichilia dregeana* resulted in MIC values ranging from 0.8 mg/mL to > 16.0 mg/mL while ∑FIC values ranged from 0.2 to 0.5, implying synergistic effects irrespective of the ratio at which these two species were combined, thus supporting the traditional method of mixing the two species as herbal medicine for syphilis in South Africa [42].

## 13. Antifungal Activities

Abubakar and Majinda [68] evaluated antifungal activities of chloroform and n-hexane extracts of heartwood of* A. adianthifolia* against* Candida albicans* using the modified agar overlay method with miconazole as the positive control. The extracts exhibited weak activities with MIQ value of >100 *μ*g which was much higher than MIQ value of 0.25 *μ*g exhibited by miconazole [68]. Similarly, Tamokou et al. (2012) [27] evaluated the antifungal activities of ethyl acetate extract and compounds aurantiamide acetate** 9, **docosanoic acid** 65, **n-hexadecanoic acid** 66, **octadecanoic acid** 67, **oleic acid** 68,** and lupeol** 86** isolated from the stem bark of* A. adianthifolia* against* Candida albicans, Candida parapsilosis, Candida lusitaniae, Candida tropicalis, Candida krusei, Candida glabrata,* and* Cryptococcus neoformans* using the broth microdilution method with nystatin as the positive control. The ethyl acetate extract and aurantiamide acetate** 9** were active against all the tested pathogens with MIC values ranging from 0.4 mg/ml to 6.3 mg/ml and 0.01 mg/ml to 0.05 mg/ml, respectively [27]. The compound lupeol** 86** was active against* Candida albicans, Candida parapsilosis, Candida lusitaniae, Candida krusei,* and* Cryptococcus neoformans* with MIC values ranging from 0.1 mg/ml to 0.4 mg/ml. A mixture of n-hexadecanoic acid** 66** and oleic acid** 68**, and a mixture of docosanoic acid** 65, **n-hexadecanoic acid** 66,** and octadecanoic acid** 67** were active against* Candida albicans, Candida lusitaniae, Candida tropicalis,* and* Cryptococcus neoformans* with MIC values ranging from 0.1 mg/ml to 0.4 mg/ml. The exhibited minimum fungicidal concentration (MFC) values were 0.8 mg/ml to 6.3 mg/ml for ethyl acetate extract, 0.8 mg/ml (compounds n-hexadecanoic acid** 66** and oleic acid** 68**), 0.2 mg/ml to 0.8 mg/ml (docosanoic acid** 65, **n-hexadecanoic acid** 66** and octadecanoic acid** 67**), 0.2 mg/ml to 0.4 mg/ml (lupeol** 86**), and 0.006 mg/ml to 0.05 mg/ml (aurantiamide acetate** 9**) [27].

## 14. Anti-Inflammatory Activities

Jäger et al. (1996) [78] evaluated anti-inflammatory activities of aqueous and ethanolic bark extracts of* A. adianthifolia *in an in vitro assay for cyclooxygenase inhibitors with indomethacin (0.5 *μ*g) as the control. The ethanolic extract of* A. adianthifolia* showed an inhibition of 69% which was higher than 66.5% inhibition exhibited by the indomethacin control. Based on these results, there might be a rationale for the ethnopharmacological claim that* A. adianthifolia* possess anti-inflammatory properties [78]. Similarly, Eldeen et al. (2005) [73] evaluated anti-inflammatory activities of aqueous, ethanol, and ethyl acetate bark and root extracts of* A. adianthifolia* using the cyclooxygenase (COX-1 and COX-2) assays. Aqueous, ethanol, and ethyl acetate bark and root extracts were active against COX-1 with inhibition percentage ranging from 61% to 90% while bark and root ethyl acetate, ethanol, and aqueous bark extracts were active against COX-2 with inhibition percentage ranging from 58% to 87% [73]. These finding support the traditional use of* A. adianthifolia* as herbal medicine for abdominal pains, back pain (lumbago), and anal wounds in Cameroon, Guinea-Bissau, and Mozambique [27, 36, 50, 60].

## 15. Antioxidant Activities

Beppe et al. (2014) [28] evaluated the antioxidant activities of aqueous leaf extracts of* A. adianthifolia* using the 1,1-diphenyl-2-picrylhydrazyl (DPPH) radical scavenging assay. The DPPH method showed total antioxidant activities of 58.2% [28]. Sonibare et al. (2017) [69] evaluated the antioxidant activities of methanol, ethyl acetate, chloroform, and n-hexane leaf extracts of* A. adianthifolia* using DPPH free radical scavenging activity assay. All the extracts showed activities with IC_50_ values ranging from 55.8 *μ*g/mL to 232.2 *μ*g/mL [69]. Tamokou et al. (2012) [27] evaluated the antioxidant activities of ethyl acetate extract and compounds aurantiamide acetate** 9** and lupeol** 86** isolated from the stem bark of* A. adianthifolia* using the DPPH free radical scavenging and trolox equivalent antioxidant capacity (TEAC) assays. Both with the DPPH and TEAC methods, compound 10 showed activities with half maximal effective concentration (EC_50_) value of 9.5 *μ*g/mL and TEAC value of 78.8 *μ*g/mL showed the highest antioxidant activity while ethyl acetate extract exhibited EC_50_ value of 70.1 *μ*g/mL and TEAC value of 46.7 *μ*g/mL [27]. Beppe et al. (2015) [79] evaluated the antioxidant activity of aqueous leaf extracts of* A. adianthifolia* using superoxide dismutase, glutathione peroxidase and catalase specific activities, the total content of the reduced glutathione, protein carbonyl, and malondialdehyde levels. The increased activities of superoxide dismutase, glutathione peroxidase, catalase, and glutathione level and the decreased levels of protein carbonyl and malondialdehyde induced by administration of the aqueous extract of* A. adianthifolia* leaves implied that this plant extract possesses strong antioxidant property [79]. Sulaiman et al. (2017) [80] evaluated the antioxidant activities of magnetic iron oxide nanoparticles synthesized using* A. adianthifolia* leaf extracts by using the DPPH free radical scavenging assay. The free radical scavenging potential of the magnetic iron oxide nanoparticles was confirmed based on its stable antioxidant effects [80].

## 16. Anxiolytic and Antidepressant Activities

Beppe et al. (2015) [79] evaluated the anxiolytic and antidepressant activities of aqueous leaf extracts of* A. adianthifolia* in the amygdala of 6-hydroxydopamine treated rats model of Parkinson's disease. The extract was administered orally to male Wistar rats at 150 mg/kg and 300 mg/kg daily for 21 days and anxiety and depression were assessed using elevated plus-maze and forced swimming tests. Administration of the extract resulted in anxiolytic and antidepressant-like effects which included a decrease of the exploratory activities, the percentage of the time spent, and the number of entries in the open arm within elevated plus-maze tests and decrease of swimming time and increase of immobility time within forced swimming test [79].

## 17. Cognitive-Enhancing Activities

Beppe et al. (2014) [28] evaluated the cognitive-enhancing activities of aqueous leaf extracts of* A. adianthifolia* in the 6-hydroxydopamine-lesion rodent model of Parkinson's disease. The extract was administered orally to male Wistar rats at 150 mg/kg and 300 mg/kg daily for 21 days and spatial memory performance was assessed using y-maze and radial arm-maze tasks. The 6-hydroxydopamine-treated rats exhibited a decrease of spontaneous alternations percentage within y-maze task and an increase of working memory errors and reference memory errors within radial arm-maze task. Administration of the aqueous extract of* A. adianthifolia* leaves significantly improved these parameters, suggesting positive effects on spatial memory formation [28].

## 18. Haemolytic Activities

Haddad et al. (2003) [66] evaluated the haemolytic activities of the crude saponin mixture, compounds** 84, 85**, and** 78** isolated from the roots of* A. adianthifolia* using the haemolysis assay against sheep erythrocytes. The crude saponin mixture exhibited good haemolytic activities with half maximal haemolytic concentration (HC50) value of 12 *μ*g/mL, while compounds 16*α*-hydroxy-21*β*-[(2-hydroxybenzoyl)oxy]-3*β*-[(O-*β*-D-xylopyranosyl-(1→2)-O-*β*-D-fucopyranosyl-(1→6)-2-(acetylamino)-2-deoxy-*β*-D-glucopyranosyl)oxy]olean-12-en-28-oic acid 28-O-*α*-L-arabinofuranosyl-(1→4)-O-[*β*-D-glucopyranosyl-(1→3)]-O-*α*-L-rhamnopyranosyl-(1→2)-*β*-D-glucopyranosyl ester** 77** and 16*α*-hydroxy-21*β*-[(2-hydroxybenzoyl)-oxy]-3*β*-[(O-*β*-D-glucopyranosyl-(1→2)-O-[O-*β*-D-xylopyranosyl-(1→2)-O-*β*-D-fucopyranosyl-(1→6)]-*β*-D-glucopyranosyl)oxy]-olean-12-en-28-oic acid 28-O-*α*-L-arabinofuranosyl-(1→4)O-[*β*-D-glucopyranosyl-(1→3)]-O-*α*-L-rhamnopyranosyl-(1→2)*β*-D-glucopyranosyl ester** 78** exhibited activities with HD_50 _values of 17.5 *μ*g/mL and 48 *μ*g/mL, respectively [66]. Similarly, Haddad et al. [81] evaluated the haemolytic activities of compounds 16*α*-hydroxy-21*β*-[(2-hydroxybenzoyl)oxy]-3*β*-[(O-*β*-D-xylopyranosyl-(1→2)-O-*β*-D-fucopyranosyl-(1→6)-2-(acetylamino)-2-deoxy-*β*-D-glucopyranosyl)oxy]olean-12-en-28-oic acid 28-O-*α*-L-arabinofuranosyl-(1→4)-O-[*β*-D-glucopyranosyl-(1→3)]-O-*α*-L-rhamnopyranosyl-(1→2)-*β*-D-glucopyranosyl ester** 77**, 16*α*-hydroxy-21*β*-[(2-hydroxybenzoyl)-oxy]-3*β*-[(O-*β*-D-glucopyranosyl-(1→2)-O-[O-*β*-D-xylopyranosyl-(1→2)-O-*β*-D-fucopyranosyl-(1→6)]-*β*-D-glucopyranosyl)oxy]-olean-12-en-28-oic acid 28-O-*α*-L-arabinofuranosyl-(1→4)O-[*β*-D-glucopyranosyl-(1→3)]-O-*α*-L-rhamnopyranosyl-(1→2)*β*-D-glucopyranosyl ester** 78,** and 21-O-{(2E,6S)-6-{{4-O-[(2E,6S)-2,6-dimethyl-6-(*β*-d-quinovopyranosyloxy)octa-2,7-dienoyl]-*β*-d-quinovopyranosyl}oxy}-2-(hydroxymethyl)-6-methylocta-2,7-dienoyl}-3-O-{O-*β*-D-xylopyranosyl-(1→2)-O-*β*-d-fucopyranosyl-(1→6)-2-(acetylamino)-2-deoxy-*β*-d-glucopyranosyl}acacic acid 28-{O-*α*-L-arabinofuranosyl-(1→4)-O-[*β*-d-glucopyranosyl-(1→3)]-O-*α*-L-rhamnopyranosyl-(1→2)-*β*-d-glucopyranosyl} ester** 80** isolated from the roots of* A. adianthifolia* using the haemolysis assay against sheep erythrocytes. The compounds 16*α*-hydroxy-21*β*-[(2-hydroxybenzoyl)oxy]-3*β*-[(O-*β*-D-xylopyranosyl-(1→2)-O-*β*-D-fucopyranosyl-(1→6)-2-(acetylamino)-2-deoxy-*β*-D-glucopyranosyl)oxy]olean-12-en-28-oic acid 28-O-*α*-L-arabinofuranosyl-(1→4)-O-[*β*-D-glucopyranosyl-(1→3)]-O-*α*-L-rhamnopyranosyl-(1→2)-*β*-D-glucopyranosyl ester** 77**, 16*α*-hydroxy-21*β*-[(2-hydroxybenzoyl)-oxy]-3*β*-[(O-*β*-D-glucopyranosyl-(1→2)-O-[O-*β*-D-xylopyranosyl-(1→2)-O-*β*-D-fucopyranosyl-(1→6)]-*β*-D-glucopyranosyl)oxy]-olean-12-en-28-oic acid 28-O-*α*-L-arabinofuranosyl-(1→4)O-[*β*-D-glucopyranosyl-(1→3)]-O-*α*-L-rhamnopyranosyl-(1→2)*β*-D-glucopyranosyl ester** 78,** and 21-O-{(2E,6S)-6-{{4-O-[(2E,6S)-2,6-dimethyl-6-(*β*-d-quinovopyranosyloxy)octa-2,7-dienoyl]-*β*-d-quinovopyranosyl}oxy}-2-(hydroxymethyl)-6-methylocta-2,7-dienoyl}-3-O-{O-*β*-D-xylopyranosyl-(1→2)-O-*β*-d-fucopyranosyl-(1→6)-2-(acetylamino)-2-deoxy-*β*-d-glucopyranosyl}acacic acid 28-{O-*α*-L-arabinofuranosyl-(1→4)-O-[*β*-d-glucopyranosyl-(1→3)]-O-*α*-L-rhamnopyranosyl-(1→2)-*β*-d-glucopyranosyl} ester** 80** exhibited haemolytic activities with HC_50_ values ranging from 12.5 *μ*g/mL to 36.6 *μ*g/mL [81].

## 19. Hypoglycemic and Antihyperglycemic Activities

Amuri et al. (2017) [45] evaluated the hypoglycemic and antihyperglycemic activities of leaf extracts of* A. adianthifolia* by administering 500 mg/kg to guinea pigs (*Cavia porcellus*), both in glucose baseline conditions and in oral glucose tolerance test with follow-up over 210 minutes. In hypoglycemic tests, the extract induced activities, lowering the normal glycemia by 33% which was comparable to the activities of the positive control, and glibenclamide (6 mg/kg) which induced a blood glucose lowering effect of 55%. In oral glucose tolerance test, the extract was active, causing inhibition of glycemia increase of 57% which was comparable to the hyperglycemic inhibition rate of glibenclamide of 50% [45]. These findings support the traditional use of* A. adianthifolia* leaf and stem bark decoction as herbal medicine for diabetes in the DRC [45] and Nigeria [29].

## 20. Immunomodulatory Activities

Haddad et al. (2003) [66] evaluated the immunomodulatory activities of the crude saponin mixture, compounds acacic acid 3-O-beta-D-xylopyranosyl-(1-->2)-beta-D-fucopyranosyl-(1-->6)- 2-acetylamino-2-deoxy-beta-D-glucopyranoside** 84**, acacic acid 3-O-(beta-D-xylopyranosyl-(1-->2)-beta-D-fucopyranosyl-(1-->6)- [beta-D-glucopyranosyl-(1-->2)]-beta-D-glucopyranosyl)-21-O-(6(S)-2- hydroxymethyl-6-methyl-6-O-(beta-D-quinovopyranosyl)-2,7-octadienoyl) ester** 85,** and 16*α*-hydroxy-21*β*-[(2-hydroxybenzoyl)-oxy]-3*β*-[(O-*β*-D-glucopyranosyl-(1→2)-O-[O-*β*-D-xylopyranosyl-(1→2)-O-*β*-D-fucopyranosyl-(1→6)]-*β*-D-glucopyranosyl)oxy]-olean-12-en-28-oic acid 28-O-*α*-L-arabinofuranosyl-(1→4)O-[*β*-D-glucopyranosyl-(1→3)]-O-*α*-L-rhamnopyranosyl-(1→2)*β*-D-glucopyranosyl ester** 78** isolated from the roots of* A. adianthifolia* using an in vitro lymphocyte proliferation assay. The cellular proliferation was measured by 3H-thymidine incorporation in Jurkat tumor cell lines (human T cell leukemia). The compounds acacic acid 3-O-beta-D-xylopyranosyl-(1-->2)-beta-D-fucopyranosyl-(1-->6)- 2-acetylamino-2-deoxy-beta-D-glucopyranoside** 84** and acacic acid 3-O-(beta-D-xylopyranosyl-(1-->2)-beta-D-fucopyranosyl-(1-->6)- [beta-D-glucopyranosyl-(1-->2)]-beta-D-glucopyranosyl)-21-O-(6(S)-2- hydroxymethyl-6-methyl-6-O-(beta-D-quinovopyranosyl)-2,7-octadienoyl) ester** 85** exhibited a dose-dependent immunomodulatory effect in the concentration range of 0.01 *μ*M to 10 *μ*M, whereas compound 16*α*-hydroxy-21*β*-[(2-hydroxybenzoyl)-oxy]-3*β*-[(O-*β*-D-glucopyranosyl-(1→2)-O-[O-*β*-D-xylopyranosyl-(1→2)-O-*β*-D-fucopyranosyl-(1→6)]-*β*-D-glucopyranosyl)oxy]-olean-12-en-28-oic acid 28-O-*α*-L-arabinofuranosyl-(1→4)O-[*β*-D-glucopyranosyl-(1→3)]-O-*α*-L-rhamnopyranosyl-(1→2)*β*-D-glucopyranosyl ester** 78** showed a lymphoproliferative activity in the concentration range of 0.01 *μ*M to 10 *μ*M [66].

## 21. Cytotoxicity Activities

Naidoo et al. (2013) [77] evaluated cytotoxicity activities of aqueous and dichloromethane and methanol (1:1) bark extracts of* A. adianthifolia* using the 3-[4,5-dimethyl-2-thiazol-yl]-2,5-diphenyl2H-tetrazolium bromide (MTT) cellular viability assay on the human embryonic kidney epithelial (Graham, HEK-293) cell line. The cell viability assay indicated that the extracts were nontoxic at 100 mg/ml against the human kidney epithelial cell line, but 110% and 112% cell growth exhibited by aqueous and organic extracts, respectively, appeared to increase cellular activity, which would be effective in wound healing [77]. Kuete et al. (2016) [84] evaluated the cytotoxicity activities of methanol bark and root extracts of* A. adianthifolia* against the sensitive leukemia CCRF-CEM cells. The extracts were further tested on a panel of eight human cancer cell lines, including MDR phenotypes. In the preliminary assay using CCRF-CEM cells, the bark and root extracts exhibited activities with IC_50_ values of 0.98 *μ*g/mL and 1.5 *μ*g/mL, respectively. Both bark and root extracts were active against other cell lines and normal AML12 hepatocytes with IC_50_ values ranging from 2.7 *μ*g/mL to 10.8 *μ*g/mL towards glioblastoma U87MG.ΔEGFR cells, breast adenocarcinoma MDA-MB-231-BCRP cells, and colon carcinoma HCT116(p53^−/−^) cells. The root extracts induced apoptosis in CCRF-CEM cells through caspases activation and loss of mitochondrial membrane potential [84]. Sulaiman et al. (2017) [80] evaluated the cytotoxic activities of magnetic iron oxide nanoparticles synthesized using* A. adianthifolia* leaf extracts on human breast (AMJ-13) and (MCF-7) cancer cells. The observed antiproliferative effects towards AMJ-13 and MCF-7 are due to cell death and inducing apoptosis. Mitochondrial membrane potential and acridine orange-propidium iodide staining assays as well as single cell and DNA gel electrophoresis analyses indicated that magnetic iron oxide nanoparticles induce cell death only by apoptosis [80].

Gengan et al. (2013) [82] evaluated cytotoxic activities of silver nanoparticles (AgNP) synthesized from aqueous leaf extracts of* A. adianthifolia* on the A549 human lung cancer cell line and normal healthy human peripheral lymphocytes using MTT, ATP, and lactate dehydrogenase assays. Viability data for A549 cells showed a 21% (10 *μ*g/ml) and 73% (50 *μ*g/ml) cell viability after 6 hours exposure to AgNPs compared to 117% (10 *μ*g/ml) and 109% (50 *μ*g/ml) cell viability of normal peripheral lymphocytes [82]. Govender et al. (2013) [83] evaluated the cytotoxicity activities of silver nanoparticles (AgNP) synthesized from aqueous leaf extracts of* A. adianthifolia* on A549 lung cells. Cell viability was determined by the MTT assay by determining cellular oxidative status, lipid peroxidation and glutathione levels, ATP concentration, caspase-3/-7, caspase-8, and caspase-9 activities, apoptosis, mitochondrial membrane depolarization (flow cytometry) and DNA fragmentation, and CD95 receptors, p53, bax, PARP-1, and smac/DIABLO [83]. The silver nanoparticles of* A. adianthifolia* caused a dose-dependent decrease in cell viability with a significant increase in lipid peroxidation, decreased intracellular lipid peroxidation and glutathione, decrease in cellular ATP, elevation in mitochondria depolarization, higher apoptosis, decline in expression of CD95 receptors, reduction in caspase-8 activity, and increases in caspase-3/-7 and caspase-9 activities; western blots showed increased expression of smac/DIABLO and increased expression of p53, bax, and PARP-1 [83]. Haddad et al. (2004) [81] evaluated the cytotoxic activities of compounds acacic acid 3-O-beta-D-xylopyranosyl-(1-->2)-beta-D-fucopyranosyl-(1-->6)- 2-acetylamino-2-deoxy-beta-D-glucopyranoside** 84**, acacic acid 3-O-(beta-D-xylopyranosyl-(1-->2)-beta-D-fucopyranosyl-(1-->6)- [beta-D-glucopyranosyl-(1-->2)]-beta-D-glucopyranosyl)-21-O-(6(S)-2- hydroxymethyl-6-methyl-6-O-(beta-D-quinovopyranosyl)-2,7-octadienoyl) ester** 85,** 16*α*-hydroxy-21*β*-[(2-hydroxybenzoyl)oxy]-3*β*-[(O-*β*-D-xylopyranosyl-(1→2)-O-*β*-D-fucopyranosyl-(1→6)-2-(acetylamino)-2-deoxy-*β*-D-glucopyranosyl)oxy]olean-12-en-28-oic acid 28-O-*α*-L-arabinofuranosyl-(1→4)-O-[*β*-D-glucopyranosyl-(1→3)]-O-*α*-L-rhamnopyranosyl-(1→2)-*β*-D-glucopyranosyl ester** 77**, 16*α*-hydroxy-21*β*-[(2-hydroxybenzoyl)-oxy]-3*β*-[(O-*β*-D-glucopyranosyl-(1→2)-O-[O-*β*-D-xylopyranosyl-(1→2)-O-*β*-D-fucopyranosyl-(1→6)]-*β*-D-glucopyranosyl)oxy]-olean-12-en-28-oic acid 28-O-*α*-L-arabinofuranosyl-(1→4)O-[*β*-D-glucopyranosyl-(1→3)]-O-*α*-L-rhamnopyranosyl-(1→2)*β*-D-glucopyranosyl ester** 78,** and 21-O-{(2E,6S)-6-{{4-O-[(2E,6S)-2,6-dimethyl-6-(*β*-d-quinovopyranosyloxy)octa-2,7-dienoyl]-*β*-d-quinovopyranosyl}oxy}-2-(hydroxymethyl)-6-methylocta-2,7-dienoyl}-3-O-{O-*β*-D-xylopyranosyl-(1→2)-O-*β*-d-fucopyranosyl-(1→6)-2-(acetylamino)-2-deoxy-*β*-d-glucopyranosyl}acacic acid 28-{O-*α*-L-arabinofuranosyl-(1→4)-O-[*β*-d-glucopyranosyl-(1→3)]-O-*α*-L-rhamnopyranosyl-(1→2)-*β*-d-glucopyranosyl} ester** 80** isolated from the roots of* A. adianthifolia* on human leukemia T-cells (Jurkat cells) and on splenocytes. The compounds 16*α*-hydroxy-21*β*-[(2-hydroxybenzoyl)oxy]-3*β*-[(O-*β*-D-xylopyranosyl-(1→2)-O-*β*-D-fucopyranosyl-(1→6)-2-(acetylamino)-2-deoxy-*β*-D-glucopyranosyl)oxy]olean-12-en-28-oic acid 28-O-*α*-L-arabinofuranosyl-(1→4)-O-[*β*-D-glucopyranosyl-(1→3)]-O-*α*-L-rhamnopyranosyl-(1→2)-*β*-D-glucopyranosyl ester** 77**, 16*α*-hydroxy-21*β*-[(2-hydroxybenzoyl)-oxy]-3*β*-[(O-*β*-D-glucopyranosyl-(1→2)-O-[O-*β*-D-xylopyranosyl-(1→2)-O-*β*-D-fucopyranosyl-(1→6)]-*β*-D-glucopyranosyl)oxy]-olean-12-en-28-oic acid 28-O-*α*-L-arabinofuranosyl-(1→4)O-[*β*-D-glucopyranosyl-(1→3)]-O-*α*-L-rhamnopyranosyl-(1→2)*β*-D-glucopyranosyl ester** 78,** and 21-O-{(2E,6S)-6-{{4-O-[(2E,6S)-2,6-dimethyl-6-(*β*-d-quinovopyranosyloxy)octa-2,7-dienoyl]-*β*-d-quinovopyranosyl}oxy}-2-(hydroxymethyl)-6-methylocta-2,7-dienoyl}-3-O-{O-*β*-D-xylopyranosyl-(1→2)-O-*β*-d-fucopyranosyl-(1→6)-2-(acetylamino)-2-deoxy-*β*-d-glucopyranosyl}acacic acid 28-{O-*α*-L-arabinofuranosyl-(1→4)-O-[*β*-d-glucopyranosyl-(1→3)]-O-*α*-L-rhamnopyranosyl-(1→2)-*β*-d-glucopyranosyl} ester** 80** exhibited cytotoxic activities on Jurkat cells, while the compounds acacic acid 3-O-beta-D-xylopyranosyl-(1-->2)-beta-D-fucopyranosyl-(1-->6)-2-acetylamino-2-deoxy-beta-D-glucopyranoside** 84** and acacic acid 3-O-(beta-D-xylopyranosyl-(1-->2)-beta-D-fucopyranosyl-(1-->6)- [beta-D-glucopyranosyl-(1-->2)]-beta-D-glucopyranosyl)-21-O-(6(S)-2- hydroxymethyl-6-methyl-6-O-(beta-D-quinovopyranosyl)-2,7-octadienoyl) ester** 85** exhibited lymphoproliferative activities on this cell type. Cytotoxic activity on Jurkat cells was observed at 10-1 *μ*M and 1 *μ*M for compound 21-O-{(2E,6S)-6-{{4-O-[(2E,6S)-2,6-dimethyl-6-(*β*-d-quinovopyranosyloxy)octa-2,7-dienoyl]-*β*-d-quinovopyranosyl}oxy}-2-(hydroxymethyl)-6-methylocta-2,7-dienoyl}-3-O-{O-*β*-D-xylopyranosyl-(1→2)-O-*β*-d-fucopyranosyl-(1→6)-2-(acetylamino)-2-deoxy-*β*-d-glucopyranosyl}acacic acid 28-{O-*α*-L-arabinofuranosyl-(1→4)-O-[*β*-d-glucopyranosyl-(1→3)]-O-*α*-L-rhamnopyranosyl-(1→2)-*β*-d-glucopyranosyl} ester** 80** and at 1 *μ*M for compounds 16*α*-hydroxy-21*β*-[(2-hydroxybenzoyl)oxy]-3*β*-[(O-*β*-D-xylopyranosyl-(1→2)-O-*β*-D-fucopyranosyl-(1→6)-2-(acetylamino)-2-deoxy-*β*-D-glucopyranosyl)oxy]olean-12-en-28-oic acid 28-O-*α*-L-arabinofuranosyl-(1→4)-O-[*β*-D-glucopyranosyl-(1→3)]-O-*α*-L-rhamnopyranosyl-(1→2)-*β*-D-glucopyranosyl ester** 77** and 16*α*-hydroxy-21*β*-[(2-hydroxybenzoyl)-oxy]-3*β*-[(O-*β*-D-glucopyranosyl-(1→2)-O-[O-*β*-D-xylopyranosyl-(1→2)-O-*β*-D-fucopyranosyl-(1→6)]-*β*-D-glucopyranosyl)oxy]-olean-12-en-28-oic acid 28-O-*α*-L-arabinofuranosyl-(1→4)O-[*β*-D-glucopyranosyl-(1→3)]-O-*α*-L-rhamnopyranosyl-(1→2)*β*-D-glucopyranosyl ester** 78** [81].

## 22. Conclusion

*Albizia adianthifolia* has been used as herbal medicine in tropical Africa for several centuries and significant breakthrough has been made in the last 40 years elucidating the phytochemical and pharmacological properties of the species. However, there are still some research gaps regarding correlating the ethnomedicinal applications of the species with the chemical compounds and pharmacological properties of the compounds and extracts of the species. Detailed studies on the pharmacokinetics, in vivo, and clinical research involving compounds isolated from* A. adianthifolia* and extracts of the species are required. The bark of* A. adianthifolia* is known to be toxic [25] and roots of the species are used as fish poison in Mozambique [85]. Similarly, in southern Cameroon, the gum from the bark of* A. adianthifolia* is used as a hunting poison and in the Central African Republic, the bark and leaves of the species are used as fish poison [86]. These reports highlight the need for detailed toxicological evaluations of both the extracts of the species as well as the compounds isolated from* A. adianthifolia* to establish the toxicity and/or any side effects that can arise when the species and its products are used as herbal medicines.

## Figures and Tables

**Table 1:** Medicinal applications of *Albizia adianthifolia* in tropical Africa.

**Medicinal use**	**Parts of the plant used**	**Country**	**References**
Antidote	Root and stem bark	Cameroon	[27]

Anthrax	Leaves	Rwanda	[44]

Aphrodisiac	Leaves and stem bark	DRC	[45]

Conjunctivitis and eye problems	Bark, leaves, leaf sap, roots and stem bark	Cameroon, Nigeria, South Africa and Swaziland	[26–29, 32, 33, 46–49]

Diabetes	Leaves and stem bark	DRC and Nigeria	[29, 45]

Epilepsy	Stem bark	Swaziland	[46, 47]

**Gastro-intestinal problems**			

Diarrhoea, dysentery and stomach ache	Bark, leaves and roots	DRC, Madagascar, Mozambique, South Africa and Tanzania	[26, 30–36]

Haemorrhoids	Leaves, roots and stem bark	Nigeria and South Africa	[29, 32]

Headache	Bark and stem bark	Cameroon and South Africa	[26, 33, 50]

Hiccup	Leaves and stem bark	DRC	[45]

Hypertension	Leaves	Togo	[37]

Love charm	Bark	South Africa	[17, 25, 26]

Memory loss and neurodegenerative disorders	Leaves and roots	Nigeria and South Africa	[32, 51]

Purgative	Bark, leaves and roots	Cameroon and South Africa	[26, 28, 31–33]

**Respiratory problems**			

Bronchitis, cough, respiratory problems and sinusitis	Bark, leaves and stem bark	Cameroon, Mozambique, Nigeria and South Africa	[25–29]

Reproductive problems in women			

Sterility and uterine problems	Bark, leaves and roots	Cameroon and Swaziland	[47, 49, 52]

**Sexually transmitted infections**			

Gonorrhoea, Sexually transmitted diseases and syphilis	Bark, leaves, roots and stem bark	Cameroon, DRC, Guinea, Nigeria, Rwanda, Swaziland and Uganda	[29, 45–47, 49, 53–55]

Gonorrhoea	Stem bark mixed with *Citrus aurantiifolia* (Christm.) Swingle fruits	Sierra Leone	[41]

Syphilis	Leaves mixed with *Trichilia dregeana* Sond. bark	South Africa	[42]

**Skin diseases**			

Abscesses, chicken pox, eczema, purulent rashes, scabies and skin diseases	Bark, leaves and roots	Burundi, Cameroon, South Africa and Swaziland	[28, 33, 44, 47, 49, 52, 56–59]

Toothache	Leaves and roots	South Africa	[32]

Typhoid fever	Stem bark	Cameroon	[27]

Urinary problems	Stem bark	Cameroon	[27]

**Wounds and pain**			

Abdominal pains, back pain (lumbago) and anal wounds	Bark, leaves and stem bark	Cameroon, Guinea-Bissau and Mozambique	[27, 36, 50, 60]

Yaws	Root bark	Rwanda	[44]

**Ethnoveterinary medicine**			

Coccidiosis and wounds	Roots	Zimbabwe	[61, 62]

Ethnoveterinary medicine	Blisters treated with leaves mixed with those of *Gynura scandens* O. Hoffm. and fruits of *Musa paradisiaca* L.	DRC	[43]

**Table 2:** Phytochemical compounds identified from *Albizia adianthifolia.*

**No.**	**Compound**	**Method of compound analyses**	**Plant part**	**References**
	**Apocarotenoids**			

**1**	*β*-cyclocitral	GC and GC-MS	Leaves	[70]

**2**	*β*-cyclohomocitral	GC and GC-MS	Leaves	[70]

**3**	cis-*α*-ambrinol	GC and GC-MS	Leaves	[70]

**4**	(E)-*α*-ionone	GC and GC-MS	Leaves and stem bark	[70]

**5**	(E)-*β*-damascenone	GC and GC-MS	Leaves	[70]

**6**	(E)-*β*-ionone	GC and GC-MS	Leaves	[70]

**7**	(E)-geranyl acetone	GC and GC-MS	Leaves	[70]

**8**	Safranal	GC and GC-MS	Leaves	[70]

	**Dipeptide**			

**9**	Aurantiamide acetate	GC-MS	Stem bark	[27]

	**Elliptosides **			

**10**	Monodesmonoterpenyl elliptoside A	NMR	Roots	[67]

	**Essential oils**			

	**Non-terpene derivatives**			

**11**	1-octen-3-one	GC and GC-MS	Root bark	[70]

**12**	1,4-dimethyltetralin	GC and GC-MS	Leaves	[70]

**13**	1,8-cineole	GC and GC-MS	Leaves, roots and stem bark	[70]

**14**	2-heptanone	GC and GC-MS	Leaves	[70]

**15**	2-octanone	GC and GC-MS	Leaves	[70]

**16**	2-pentadecanone	GC and GC-MS	Root bark	[70]

**17**	2-phenylethyl butanoate	GC and GC-MS	Root bark	[70]

**18**	2,4-dimethylbenzaldehyde	GC and GC-MS	Leaves	[70]

**19**	5-methyltetralin	GC and GC-MS	Leaves	[70]

**20**	6-methyl-5-hepten-2-one	GC and GC-MS	Leaves	[70]

**21**	*α*-ionone	GC and GC-MS	Leaves	[70]

**22**	Benzaldehyde	GC and GC-MS	Root bark	[70]

**23**	Decanal	GC and GC-MS	Leaves	[70]

**24**	Dehydro-ar-ionene	GC and GC-MS	Leaves	[70]

**25**	Mesitylene	GC and GC-MS	Root bark	[70]

**26**	Naphthalene	GC and GC-MS	Leaves and root bark	[70]

**27**	Nonanal	GC and GC-MS	Leaves	[70]

**28**	Pentadecanal	GC and GC-MS	Leaves, roots and stem bark	[70]

**29**	n-tetradecane	GC and GC-MS	Leaves	[70]

**30**	n-tridecane	GC and GC-MS	Leaves	[70]

**31**	Seudenone	GC and GC-MS	Leaves	[70]

**32**	Tetradecanal	GC and GC-MS	Roots and stem bark	[70]

**33**	(Z,E)-undeca-1,3,5-triene	GC and GC-MS	Leaves	[70]

	**Oxygenated monoterpenes**			

**34**	*α*-terpineol	GC and GC-MS	Leaves	[70]

**35**	Linalool	GC and GC-MS	Leaves and root bark	[70]

**36**	p-menth-4-en-3-one	GC and GC-MS	Leaves	[70]

**37**	Thymol	GC and GC-MS	Stem bark	[70]

	**Oxygenated sesquiterpenes**			

**38**	Acorenone	GC and GC-MS	Leaves and stem bark	[70]

**39**	Caryophylla-4(14),8(15)-dien-5-ol	GC and GC-MS	Leaves and stem bark	[70]

**40**	Caryophyllene oxide	GC and GC-MS	Leaves, roots and stem bark	[70]

**41**	(E)-nerolidol	GC and GC-MS	Leaves and stem bark	[70]

**42**	Humulene epoxide II	GC and GC-MS	Roots and stem bark	[70]

**43**	Occidentalol	GC and GC-MS	Root bark	[70]

**44**	Selin-11-en-4-*α*-ol	GC and GC-MS	Roots and stem bark	[70]

**45**	T-cadinol	GC and GC-MS	Leaves and stem bark	[70]

**46**	trans-*β*-elemenone	GC and GC-MS	Stem bark	[70]

**47**	Valerianol	GC and GC-MS	Root bark	[70]

**48**	Viridiflorol	GC and GC-MS	Leaves, roots and stem bark	[70]

	**Sesquiterpene hydrocarbons**			

**49**	*α*-bulnesene	GC and GC-MS	Stem bark	[70]

**50**	*α*-humulene	GC and GC-MS	Leaves, roots and stem bark	[70]

**51**	ar-curcumene	GC and GC-MS	Leaves and stem bark	[70]

**52**	*α*-zingiberene	GC and GC-MS	Leaves and stem bark	[70]

**53**	*β*-bisabolene	GC and GC-MS	Stem bark	[70]

**54**	*β*-caryophyllene	GC and GC-MS	Leaves, roots and stem bark	[70]

**55**	*β*-elemene	GC and GC-MS	Stem bark	[70]

**56**	*β*-sesquiphellandrene	GC and GC-MS	Leaves and stem bark	[70]

**57**	Cyperene	GC and GC-MS	Root bark	[70]

**58**	*δ*-cadinene	GC and GC-MS	Root bark	[70]

**59**	Isocaryophyllene	GC and GC-MS	Stem bark	[70]

**60**	Italicene	GC and GC-MS	Leaves	[70]

**61**	Sesquisabinene	GC and GC-MS	Stem bark	[70]

**62**	trans-*γ*-cadinene	GC and GC-MS	Stem bark	[70]

**63**	*γ*-muurolene	GC and GC-MS	Stem bark	[70]

	**Fatty acid**			

**64**	9,12-octadecadienoic acid (Z,Z)-,methyl ester	GC-MS	Heartwood	[68]

**65**	Docosanoic acid	GC-MS	Stem bark	[27]

**66**	n-hexadecanoic acid	GC-MS	Heartwood and stem bark	[27, 68]

**67**	Octadecanoic acid	GC-MS	Stem bark	[27]

**68**	Oleic acid	GC-MS	Heartwood and stem bark	[27, 68]

**69**	trans-13-octadecanoic acid, methyl ester	GC-MS	Heartwood	[68]

	**Flavonoids**			

**70**	3-methoxyflavanone	MS	Heartwood	[64]

**71**	Apigenin	HPLC	Leaves	[28]

**72**	Chalcone (okanin)	MS	Heartwood	[64]

**73**	Melanoxetin	MS	Heartwood	[64]

	**Steroids**			

**74**	Chondrillasterol	GC-MS	Heartwood	[68]

**75**	Stigmasterol	GC-MS	Heartwood	[68]

**76**	24S,5*α* stigmast-7-en-3*β*-ol	GC-MS	Heartwood	[68]

	**Triterpene saponins**			

**77**	Adianthifoliosides A	FABMS, HRESIMS and NMR	Roots	[66]

**78**	Adianthifoliosides B	FABMS, HRESIMS and NMR	Roots	[66]

**79**	Adianthifoliosides C	NMR	Roots	[67]

**80**	Adianthifoliosides D	NMR	Roots	[67]

**81**	Adianthifoliosides E	NMR	Roots	[67]

**82**	Adianthifoliosides F	NMR	Roots	[67]

**83**	Julibroside A3	NMR	Roots	[65, 67]

**84**	Prosapogenin 1	FABMS, HRESIMS and NMR	Roots	[65, 66]

**85**	Prosapogenin 2	FABMS, HRESIMS and NMR	Roots	[65, 66]

	**Triterpenoids**			

**86**	Lupeol	GC-MS	Stem bark	[27]

**87**	3*β*e,16*β*e-dimethoxyolean-12-en-28-21*β*a-olide	NMR	Root bark	[63]

	**Histamine**			

**88**	Histamine	GC-MS	Root and stem bark	[71]

**89**	Acetylhistamine	GC-MS	Root and stem bark	[71]

	**Imidazolyl carboxylic acid**			

**90**	Imidazoleacetic acid	GC-MS	Root and stem bark	[71]
